# A Coupled Eulerian-Lagrangian Simulation and Tool Optimization for Belt Punching Process with a Single Cutting Edge

**DOI:** 10.3390/ma14185406

**Published:** 2021-09-18

**Authors:** Dominik Wojtkowiak, Krzysztof Talaśka, Dominik Wilczyński, Jan Górecki, Krzysztof Wałęsa

**Affiliations:** Institute of Machine Design, Faculty of Mechanical Engineering, Poznan University of Technology, Piotrowo 3, 60-965 Poznań, Poland; krzysztof.talaska@put.poznan.pl (K.T.); dominik.wilczynski@put.poznan.pl (D.W.); jan.gorecki@put.poznan.pl (J.G.); krzysztof.walesa@put.poznan.pl (K.W.)

**Keywords:** punching, belt perforation, piercing punch, multilayer polymer composite belt, tool optimization, finite element method, modeling and simulation

## Abstract

The objective of this paper is to analyze the belt punching process with the use of a single cutting edge and discuss the influence of geometrical features of the piercing punch on the perforation force. Two basic shapes of the piercing punch with a single cutting edge were tested: tools with the blade pointing inside or pointing outside. The analytical models of the stress distribution in the shearing cross sections were derived for both punches. The presented model, along with the series of empirical tests and Coupled Eulerian-Lagrangian simulation, was used for finding the effective geometry of the piercing punch with a single cutting edge for the belt perforation. The geometrical parameters taken into consideration for the tool optimization were the following: angle of the blade, thickness of the wall and diameter of the piercing punch cutting edge. The obtained results show that changing these parameters has a significant influence on the perforation force necessary to execute the machining process and affects the quality of the holes in the perforated belts. The most important geometrical features of the hollow sharpened punch are the angle and the direction of the blade, which change the force distribution and, as a result, the mechanics of the process.

## 1. Introduction

**Figure 1:**
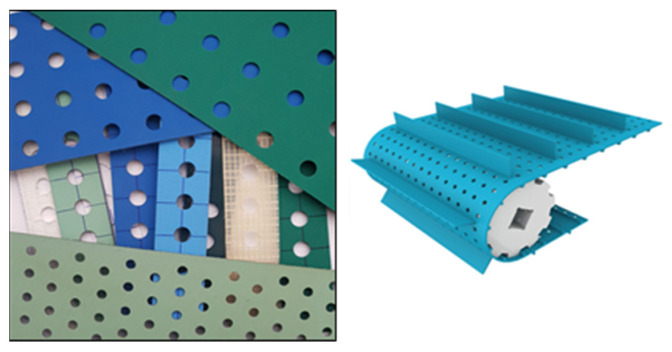
Multilayer polymer composite perforated belts used in vacuum belt transportation [3].

A perforated belt (Figure 1) is a conveyor with a pattern of punched holes selected based on its application. It is mostly used for vacuuming purposes to effectively hold products (such as boards, foils, panels, cartons, papers, etc.) during transportation. It can be also used for drying, since the holes allow air to flow around the products [1]. Due to the variety of mechanical properties of the multilayer polymer composite belts used in vacuum belt conveyors, a lot of technical issues can arise during the punching process. Adjusting the manufacturing process for perforated belts to a certain type of belt requires selecting the effective tool (considering its geometry and material properties) and proper technological parameters, such as punch velocity or temperature [2].

As was proved in [4], punching is the most suitable method for precision perforation of belts. One can distinguish punching with a single cutting edge (the hollow piercing punch which cooperates with a reducer plate) or with two cutting edges (the piercing punch and the die). Both methods have their pros and cons and are suitable for different types of multilayer polymer composite belts. Compared with the other method, punching with a single cutting edge eliminates the need to align the punch and the die. This is significant, since maintaining a very small punch-die clearance, which is required for the polymer multilayer composite belts, generates a lot of technical issues for designers of punching machines. Additionally, the sharpness of the tool, combined with the support of the belt, increases the quality of the holes by decreasing the number of uncut fibers, especially for light elastic belts [5]. On the other hand, using hollow punches generates problems with scrap utilization since it requires the mechanisms of pushing the scrap from the punch and from the surface of the belt. It can be done either mechanically or pneumatically. Since the reducer plate is made of quite an elastic material, such as polyamide PA6 or polypropylene, its deflection has to be taken into consideration to adjust the stroke of the punch in order to fully cut through the material. Different types of belts require different penetration values of the cutting edge in the reducer plate, which results in a more complex steering process or in reducing the tool life if the penetration value is set as constant with a surplus [5]. In this paper, the authors have focused on belt punching with a single cutting edge.

**Figure 2:**
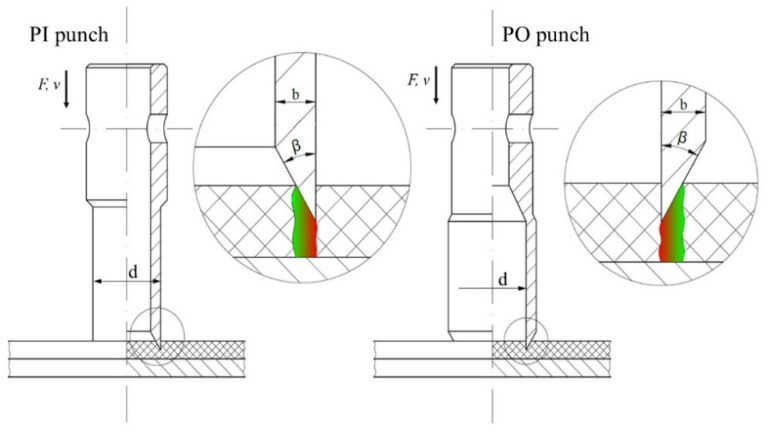
Schematic of the belt perforation process with single cutting edge.

When it comes to piercing punches which cooperate with the reducer plate, one can distinguish two basic types of tools: with the cutting edge pointing inside (PI) or pointing outside (PO). In both cases, one can distinguish three main geometrical parameters which should be taken into consideration during the analysis of the tool performance: the angle of the blade β, the thickness of the wall b and the diameter of the piercing punch cutting edge d (Figure 2).

Comparing the performance of both tools, the main difference is the deformation of the punched belt. In case of the PO punch, the final product is deformed, while in case of the PI punch, the scrap is the deformed part. It may affect the diameter of the punched holes—it will be slightly bigger than nominal value in the first case and slightly lower in the second one. Depending on the application of the perforated belt, it may be treated as an advantage or disadvantage—for mounting or positioning, too much clearance may be problematic, while two small holes may not be able to generate effective holding in vacuum transport. If the distance between the neighboring holes in the perforated pattern is small, the deformation of the perforated belt may cause a decrease in the belt strength. Additionally, it is harder to compress the scrap than extend the belt, which may result in increasing the frictional drag between the tool and the belt material and, as a result, cause the growth of the perforation force.

Comparing the design features of both tools, it should be mentioned that a slightly greater diameter of the raw material is necessary for the PO punch, since the working part of the punch is greater than the gripping part (as opposed to the PI punch). As a result, the thickness of the wall can be greater for such a tool, because it is not restricted by the inside diameter of the hollow punch (which is related to the size of the scrap pushing device). The complexity of the construction of both tools is very similar, however; due to the technological issues with the size of the grinding tool, it is easier to machine the punch with the cutting edge pointing outside.

Based on the above theoretical analysis, it is hard to clearly state which of these two punches will be more effective for perforation of multilayer polymer composite belts. Additionally, only a few research papers which discuss similar tools can be found [6,7], and they are connected more with medicine rather than mechanical engineering. For that reason, the authors decided to conduct a series of experimental tests and computer simulations in order to present the influence of geometrical parameters of piercing punches on the perforation force and the stress level in the punch. The methodology of these tests and simulations is presented in Section 2. Based on the experimental results, which are presented in Section 3.1, the validation of the FEM model has been made. The validation itself is discussed in Section 3.2, while the results of the application of the model developed in this way are presented in Section 3.3. Based on the obtained characteristics, the optimization of the piercing punch geometry was performed and the effective tool for belt punching with a single cutting edge was determined. The optimization process is described in Section 4, which is followed by the conclusions and summary in Section 5.

## 2. Materials and Methods

The conducted research was divided into two stages: experimental testing and FEM analysis.

### 2.1. Experimental Testing Methodology

**Figure 3:**
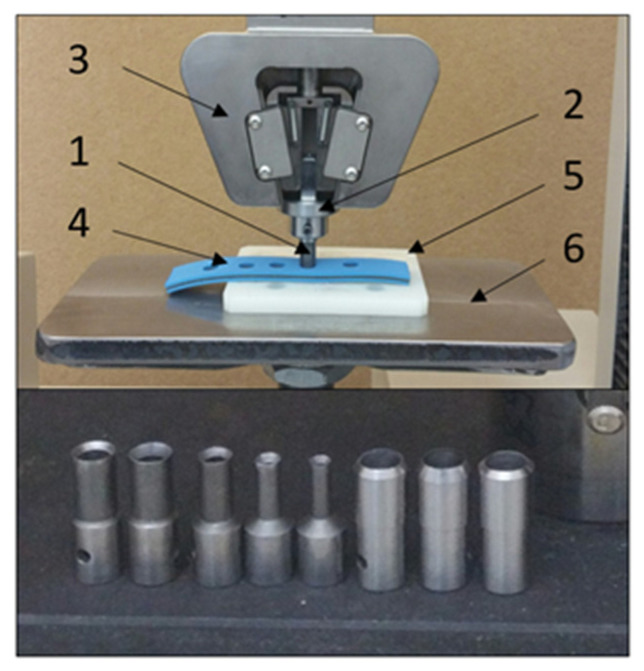
Test stand used to measure the perforation force along with the set of tools used in the experiments: 1—piercing punch, 2—punch chuck, 3—strength testing machine gripper, 4—belt specimen, 5—reducer plate, 6—base.

In the first stage of the research, the experimental tests of measuring the perforation force for various tool geometries were conducted. To obtain the results, the test stand presented in Figure 3 was used in combination with the strength testing machine MTS Insight 50 kN. The piercing punch (1) was mounted in the punch chuck (2) and blocked with a pin. The punch chuck was held by the strength testing machine upper gripper (3). The belt specimen (4) lay on the reducer plate (5), which was supported by the base plate (6) mounted to the bottom handle of the strength testing machine. During each test, values of the force and the displacement of the piercing punch were measured and, as a result, the force-displacement characteristics *F_P_*(*x*) were derived. Repetitions of the measurements were conducted five times for each type of the tool and its specific geometry.

**Figure 4:**
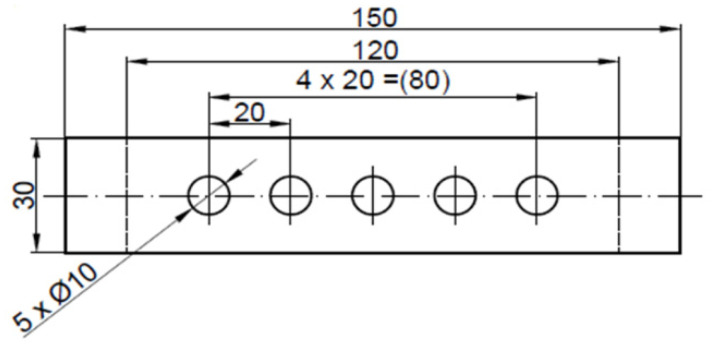
Belt specimen dimensions (in mm).

**Figure 5:**
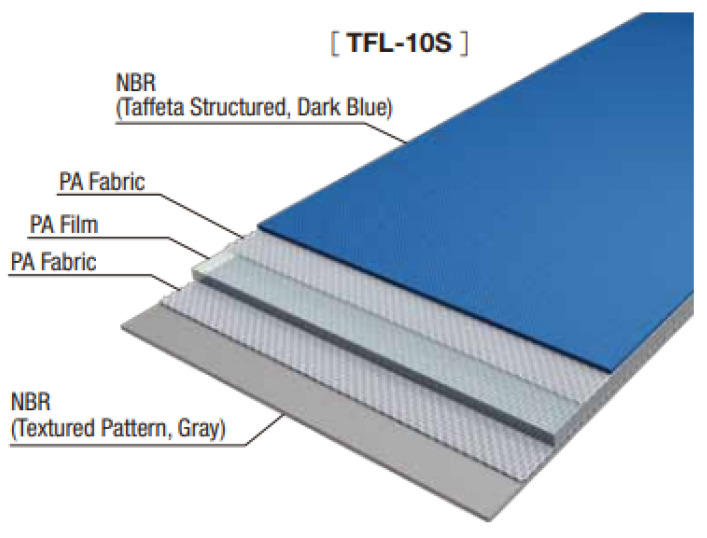
TFL10S belt (Nitta^®^ Polybelt^TM^).

The belt specimen dimensions are presented in Figure 4. Regardless of the punch diameter, the spacing between neighboring holes was equal to 20 mm, which is twice the size of the biggest diameter of the punched holes. The main belt used in the research was a rigid belt with increased strength TFL10S (Nitta, Frankfurt, Germany) (Figure 5), which consisted of five layers—1 mm thick polyamide film core in the middle, two protective gaskets made of the polyamide fabrics and the NBR rubber layers on top and at the bottom that play the role of the load-carrying and return covers. The total thickness of the belt was 2.65 mm. This type of belt was selected because it represents the group of belts which are the hardest to punch through and require the highest value of perforation force. Additionally, due to the variety of the structure of each of its layers, it is sensitive to the tool sharpness, and the quality of the holes can be easily evaluated since almost every possible defect can occur during punching [8]. To verify the trend in the obtained results, the same tests were repeated for a light elastic polyurethane belt with polyester fabric reinforcement—LAB12E (Nitta, Frankfurt, Germany), which is characterized by extremely different mechanical properties.

**Figure 6:**
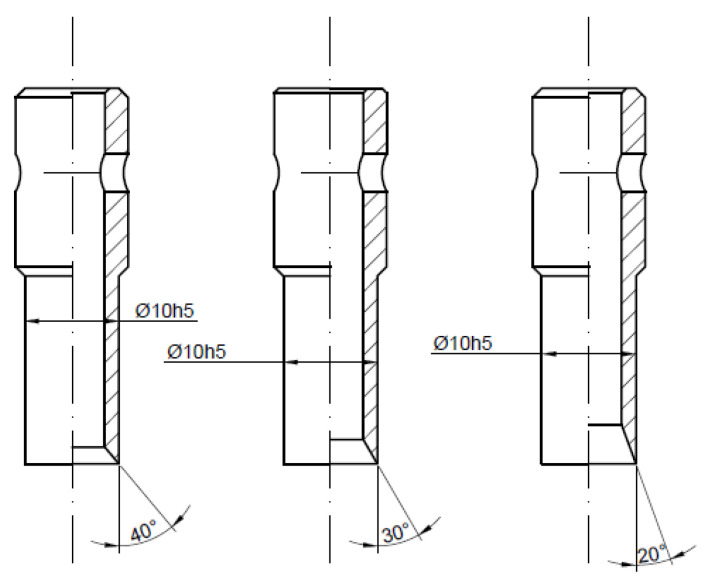
First series of tested tools—piercing punches with the cutting edge pointing towards the inside with a constant diameter and variable angle of the blade.

**Figure 7:**
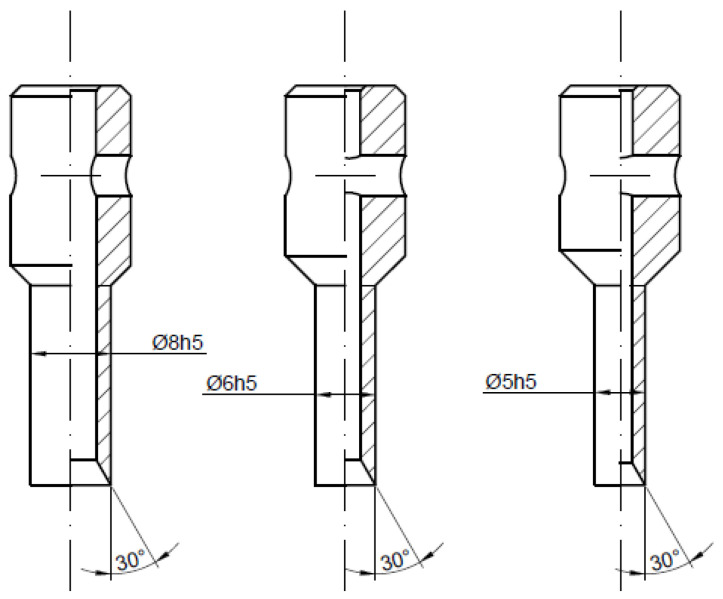
Second series of tested tools—piercing punches with the cutting edge pointing towards the inside with a variable diameter and constant angle of the blade.

**Figure 8:**
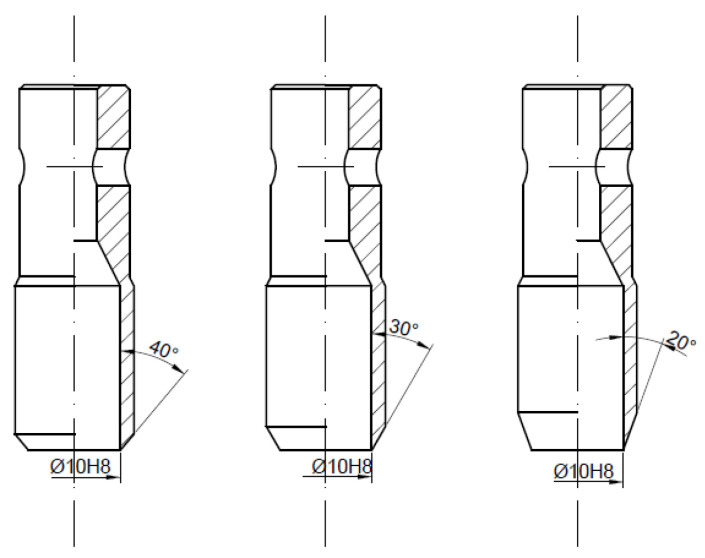
Third series of tested tools—piercing punches with the cutting edge towards the outside with a constant diameter and variable angle of the blade.

In order to determine the influence of the angle of the blade *β* and the diameter of the piercing punch cutting edge d for both types of punches (PO and PI), a set of tools made of high-speed steel SW7M was designed and manufactured. The first series of tools was PI punches where the nominal diameter d was equal to 10 mm and the variable angle of the blade *β* was equal to 20°, 30° or 40° (Figure 6). The second one represents the same type of punch but with smaller diameters *d* (5, 6 and 8 mm) with the same angle of the blade *β* equal to 30° (Figure 7). The last series consisted of three PO punches with the same parameters as the first one (Figure 8). To maintain the quasi-static characteristic of the tool performance, the test was performed with the punch velocity *v* equal to 0.5 mm per second. As a result of that approach, the influence of the process dynamics is eliminated and one can focus only on the influence of the geometrical parameters.

### 2.2. Construction of the FEM Model

In order to perform the optimization process without needing to manufacture multiple piercing punches with various tool geometries, computer simulations based on the FEM analysis can be used. Additionally, this approach eliminates the influence of the machining precision on the obtained results, which can have a crucial impact on the experimental test results. In the literature, multiple examples of FEA for punching [8,9,10,11], drilling [12,13], milling [14,15] or cutting processes [16,17] can be found.

Using the classical finite elements methods based on a Lagrangian formulation to model the punching process causes mesh element distortion, which leads to inaccuracy or aborting the calculations at early stages [18]. To avoid such a problem, the Eulerian formulation could be used, but it is not easily adaptable for modelling the unconstrained flow of the material, and therefore needs a predefined shape of the waste material [19]. To ensure a more robust analysis, the combination of the Lagrangian and the Eulerian approach can be used, e.g., the Arbitrary Lagrangian–Eulerian (ALE) or Coupled Eulerian–Lagrangian (CEL) methods [20]. Both methods make it possible to mesh the parts subjected to large deformation using the Eulerian technique, while the remaining components are treated as Lagrangian bodies. The difference between ALE and CEL methods occurs in the second step in which a new mesh is generated (the remeshing response rezoning step). In ALE, a new distinct mesh is generated, while in CEL, the rezoned mesh is the same as the original one. Both methods are suitable for modelling the material forming processes like drilling [12], cutting [16], turning [21] or extrusion [22], but the CEL method was proved as more natural in case of material behavior [12]. For that reason, the CEL method was used in the presented research.

**Figure 9:**
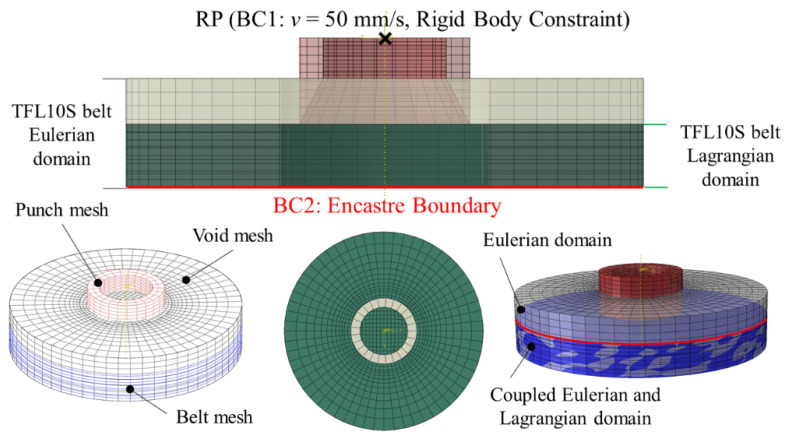
The construction of the FEM model in Abaqus using CEL method.

**Figure 10:**
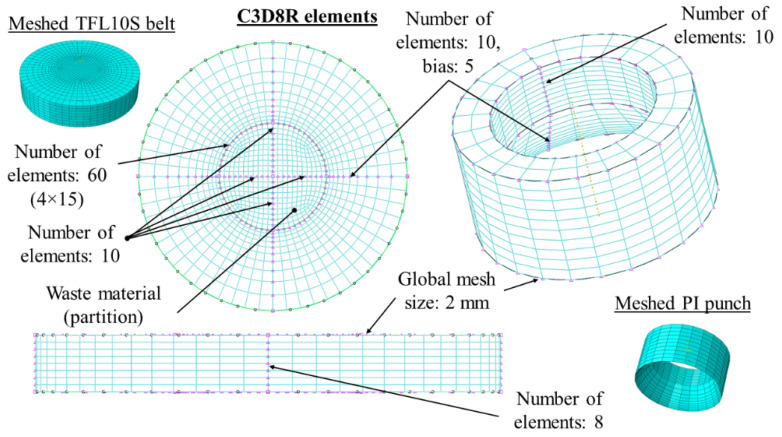
Parameters of the mesh used in FEM analysis.

The construction of the FEM model developed in Abaqus/Explicit software is presented in Figure 9. The model consists of two main instances: the punch (with various blade angles *β* in the range 15–40°, various diameters of 5, 6, 8 and 10 mm and various heights of the blade H in the range 0.5–2.65 mm for the PI punch only) and the belt. The material of the belt is defined using the parameters presented in Table 1 [11]. Using the Johnson–Cook model for simulation of the material failure had already been justified in [8,11,23,24,25,26,27]. The plasticity of the material is defined using the Yield Point of 120 MPa for the PI punch and 80 MPa for the PO punch. The belt instance is modelled as a CEL part. The Lagrangian domain is 1 mm thicker than the real belt to enable modelling the process until the punch penetrates the whole thickness of the belt and therefore obtains the most accurate force value. The Eulerian domain is thicker than the Lagrangian one, leaving the void mesh above the belt because the material of the belt will lift up slightly. Both domains are coupled with common bottom and side surface of the belt—they are overlapping. The mesh applied to the FEM model parts are presented in Figure 10. The punch was modelled as steel rigid body with Youngs’ modulus *E* = 210 GPa and 0.33 Poisson ratio. The frictional contact between the punch and the belt was defined by the friction coefficient *μ* = 0.6 in the tangential direction and “Hard” contact in the normal one. The kinematic extortion was applied to the reference point and the velocity 50 mm/s in the vertical axis was applied. The bottom surface of the belt was fixed. The mesh was steered in such a way as to provide finer mesh in the contact area between the punch and the belt. The analysis lasted 0.55 s, during which the punch moved 2.75 mm (more than the thickness of the belt). Although the velocity differed from the experimental one, it was checked that it does not affect the results significantly, but greatly reduces the computational time. The simulation parameters mentioned above are summarized in Table 2.

## 3. Results and Discussion

The experimental test results presented at the beginning of this section were used to find the influence of the geometrical parameters of the piercing punches on the perforation force and define the trends of these correlations. The obtained data helped to narrow down the range of the design variables in which the effective solution is located. Later, these data were used to validate the FEM model by which the simulation results presented at the end of this section were obtained. On their basis, the optimization process can be performed.

### 3.1. Experimental Test Results

**Figure 11:**
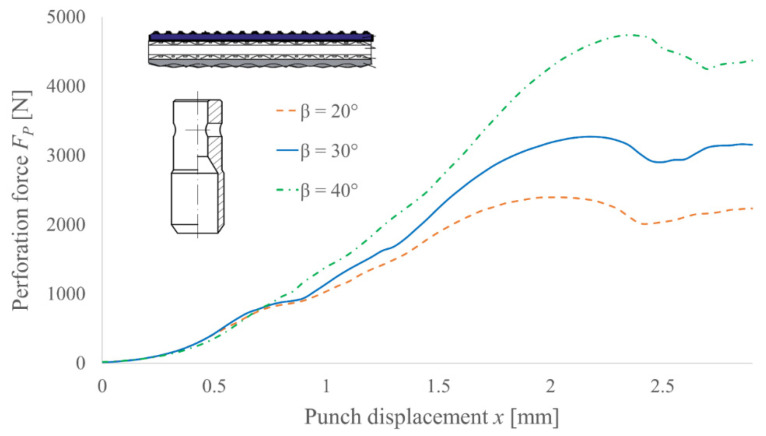
Experimental results of the perforation force measurement for PO punches with diameter *d* = 10 mm and various angles of the blade *β* for TFL10S belt.

The obtained results are presented as the perforation force in function of the punch displacement characteristics. Each of these curves is the averaged characteristic obtained from five measurements. In Figure 11, the results for various angles of the blade *β* and the constant diameter *d* = 10 mm of the PO punch for TFL10S belt are presented. On their basis, the positive trend with a supposedly linear correlation between the angle *β* and the perforation force is visible. Additionally, one can observe that all three characteristics are consistent in the beginning range of motion (below *x* = 0.8 mm, which corresponds to the depth on which the polyamide core starts). It proves that the core of the belt determines the force necessary to pierce the belt.

**Figure 12:**
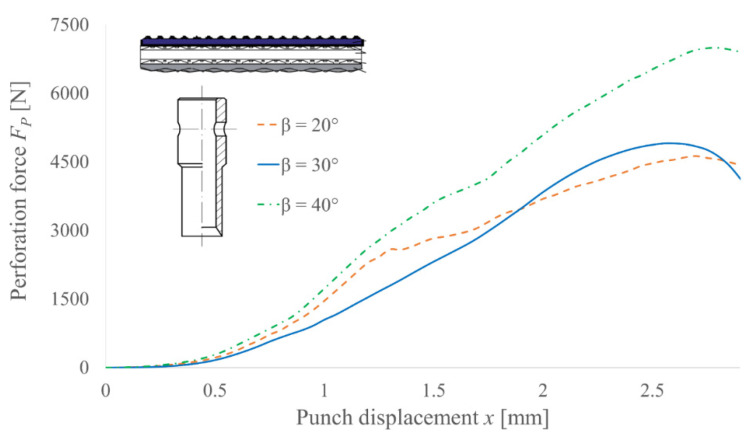
Experimental results of the perforation force measurement for PI punches with diameter *d* = 10 mm and various angles of the blade *β* for TFL10S belt.

Similar tests were performed for PI punches and their results are presented in Figure 12. In this case, the general trend is still visible, but the correlation is not so obvious. Since the consistency between all characteristics is barely visible in such a case, it follows that for this type of punch, the covers of the belt also have an impact on the perforation force. It is connected with the scrap compression inside the punch, generating much more frictional resistance on the contact surface than extending the belt, which occurs for PO punches. The irregular shape of the function for blade angle 20° also suggests that the force value depends more on the machining precision or the blade condition for this tool type.

**Figure 13:**
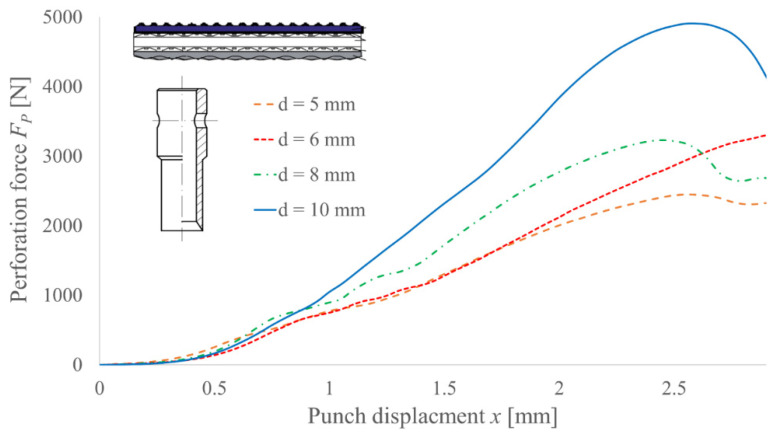
Experimental results of the perforation force measurement for PI punches with angle of the blade *β* = 30° and various diameters d for TFL10S belt.

To determine the influence of the cutting edge diameter d on the perforation force value, four PI-type punches with diameters 5, 6, 8 and 10 mm and the same angle of the blade *β* = 30° were tested; the obtained results are presented in Figure 13. As can be observed, the force reduces its value for a smaller diameter of the cutting edge. It is mainly the effect of a smaller shearing section, which suggests that a lower force is necessary to obtain the same shearing stress. However, since the complex state of stress occurs (additional compression), the divergence is nonlinear. This means that changing the angle of the blade for various diameters of the cutting edge may provide different percentages of force reduction [22].

The summary of the experimental results are presented in Table 3. Although the percentage change in the diameter of the punch and the perforation force seems to be linear for diameters 5 and 6 mm (e.g., decreasing the diameter by 50%—from 10 mm to 5 mm—reduces the force by 50% as well), some nonlinearity for the 8 mm punch can be observed (decreasing the diameter by 20% reduced the force by 34%). Such nonlinearity is the result of the machining precision, which causes force value errors of even up to 15% [11]. If one analyze the influence of the blade angle *β* solely it also suggest some nonlinearity, but in some cases (e.g., reducing the blade angle by 50%—from 40° to 20°—reduced the force by half as well). This suggests that experimental results can only show the trend of the influence. To fully discover the correlation between geometrical features and punching force, FEM analyses could be used.

### 3.2. Validation of the FEM Model

**Figure 14:**
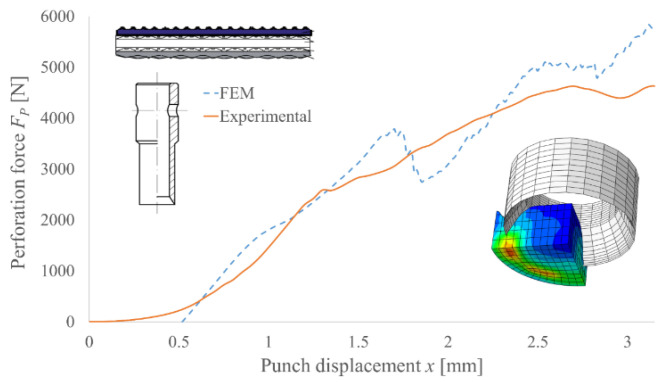
Comparison of the experimental and FEM analysis results of the perforation force for PI punch with diameter *d* = 10 mm and angle of the blade *β* = 30° for TFL10S belt.

**Figure 15:**
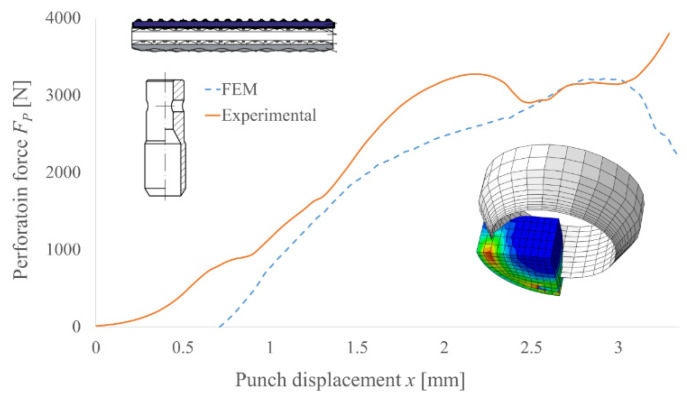
Comparison of the experimental and FEM analysis results of the perforation force for PO punch with diameter *d* = 10 mm and angle of the blade *β* = 30° for TFL10S belt.

To verify the accuracy and precision of the FEM model, the experimental results for the selected punch geometry were compared with the simulation results. The examples of such comparison for PI and PO punches are presented in Figure 14 and Figure 15, respectively. Due to the application of a few simplifications in the developed model, it is necessary to take into consideration the deformation of the polyamide reducer plate, which was neglected in the FEM model. Additionally, using the isotropic homogenous material with average mechanical properties, replacing the polymer multilayer composite belt will also require the results to be scaled to their real values. However, this approach to modelling such structures in order to determine the peak force necessary to perform the machining process is justified [8,11,24,25,26,27].

**Figure 16:**
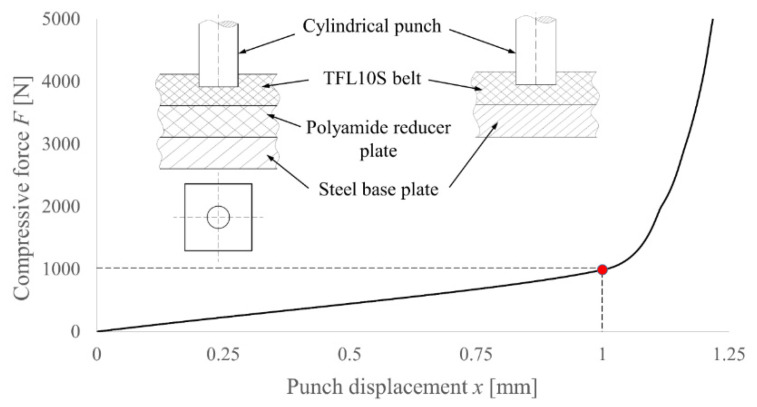
Behavior of the polyamide PA6 reducer plate under compressive load of the punch with diameter *d* = 10 mm.

To determine the deformation of the polyamide reducer plate, two compression tests of the TFL10S belt were performed. For both tests, the 10 mm cylindrical punch was used, but in the first one, the belt was lying on the steel plate, while in the second one, the reducer plate was embedded in between. Assuming that the steel plate is much more rigid than the rest of the parts, the behavior of the reducer plate was determined by subtracting the obtained results. The methodology and the results of this test are presented in Figure 16.

As can be observed, after achieving the punch displacement of 1 mm, the reducer plate started to behave almost like a rigid body. Taking into consideration the ratio of the PI punch compression area to the area of a cylinder with the same diameter, the force that will generate the same stress level can be determined and, as a result, the deformation of the reducer plate which needs to be applied to move the FEM results and adjust it to the experimental one can also be determined. To determine the compression area of the PI or PO punch, one can use the following equation:

(1) A’(x,β,d)=π · x · tan β (d ± x · tan β)

“+” is used for the PO punch and “−“ is used for the PI punch. For example, for the PI punch with diameter *d* = 10 mm and *β* = 30°, the compression area equals 0.518, which gives us a force of 518 Newtons and, as a result, the deformation of the reducer plate is 0.518 mm.

For a smaller diameter of the punch, the end of the linear range (red point) will occur for a lower force, but for a greater displacement, the proportional factor is the ratio of the diameters of the punches. For example, for a diameter of 8 mm, a force of 800 N will cause a deformation of the reducer plate of 1.25 mm, etc.

In case of the PO punches, to obtain reliable data, one must determine the multiplication factor *ξ*, which will cover the differences between tensile and compressive strain in the belt modelled as an isotropic one. Since the ratio of the compressive to tensile strain for TFL10S belt equals 4.46 (it was tested experimentally), for the sharpest analyzed punch (*β* = 15°), one must multiply the displacement of the punch by the calculated amount. On the other hand, by increasing the blade angle, an increase in the tensile strain of the belt elements occurs, which means the damage will occur faster—we need to adjust the multiplication factor ξ. Assuming that a 45° blade angle starts to compress the finite elements exactly in the same grade as it extends them, the *ξ* factor should tend to 1. Assuming a linear correlation, it is possible to determine the *ξ* factor for any blade angle within the range 15–40° using the following equation:

(2) ξ=−0.1356 · β+6.494

**Figure 17:**
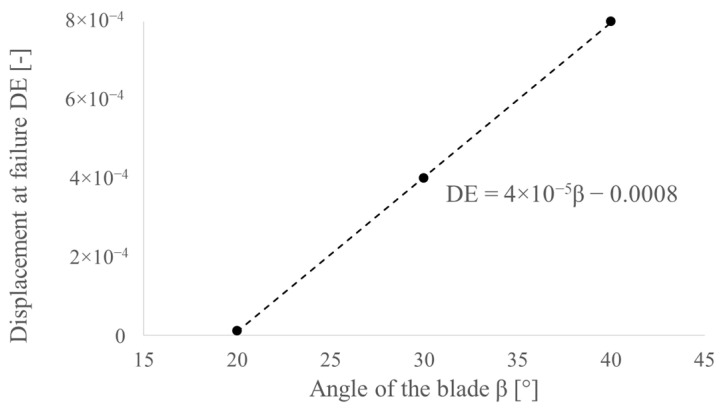
Determination of the damage evolution parameter DE (displacement at failure) for PO punches.

Additionally, to avoid element deletion that is too fast, it is necessary to adjust the damage evolution parameter DE—displacement at failure used in the Johnson–Cook damage model. Based on the experimental results, the value of DE was selected in such a way that the plastic flow of the material which occurs after achieving the peak perforation force for both experimental and simulation results will be consistent (Figure 14). The obtained correlation is presented in Figure 17.

The last aspect which needs to be discussed is the false growth of the force at the end of the analysis after plastic flow occurs (Figure 14). It is caused by the false boundary condition restraining all degrees of freedom of the bottom part of the cut belt, which in fact lies freely on the reducer plate. Additionally, the Lagrangian instance, which represents the belt, was extended in thickness by 1 mm in order to properly mirror the real conditions until the end of the punching. This means that for a displacement for which in reality the scrap is already separated from the belt, the force still rises.

The model validated in this way is suitable for performing the FEM analysis for any blade angle *β*, cutting edge diameter d or height of the blade *H*.

### 3.3. FEM Analysis Results

**Figure 18:**
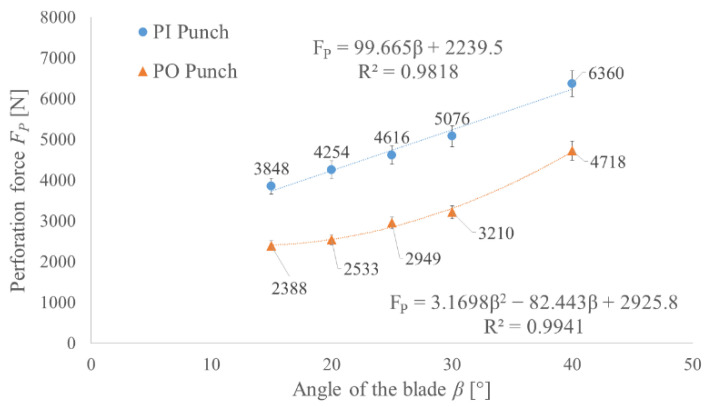
The influence of the angle of the blade *β* on the perforation force *F_P_* for both PI and PO punches with diameter *d* = 10 mm and height of the blade *H* = 2.65 mm for belt TFL10S.

Based on the developed and validated FEM model, the values of the perforation forces for both types of tools were determined. For each type, five different angles of the blade *β* were tested with a constant height of the blade *H* equal to the thickness of the TFL10S belt. The obtained results made it possible to determine the functions which can be used to estimate the force value for any angle of the blade in the tested range 15–40° with an error less than 5% (Figure 18).

In order to gain the lowest possible perforation force (minimum force criterion), the angle of the blade should be minimized, but it will cause a small thickness of the wall of the hollow punch if we assume the constant height of the blade. In order to take into consideration the durability of the punch (minimum stress criterion), the compressive stress in the punch was determined as the peak perforation force divided by the punch cross section. To determine the compressed section of the punch, the depth of the penetration *x* in Equation (1) has to be replaced with the height of the blade *H* = 2.65 mm. Increasing the blade angle will reduce the stress level in the punch, as presented in Figure 18; however, it will also increase the perforation force. Since both criteria (the minimum force and minimum stress criteria) represent opposite correlations to the change of the angle of the punch blade, it is necessary to perform an optimization process to find the effective solution.

**Figure 19:**
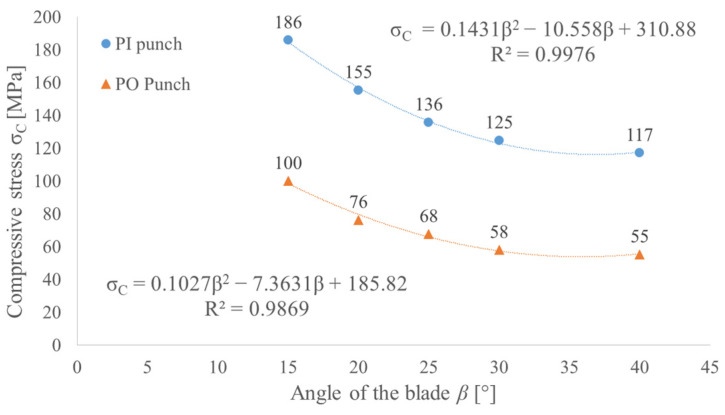
The influence of the angle of the blade *β* on the punch compressive stress *σ_C_* for both PI and PO punches with diameter *d* = 10 mm and height of the blade *H* = 2.65 mm for belt TFL10S.

As follows from the obtained results (Figure 18), by steering the value of the blade angle *β*, it is possible to reduce the force by 40% for the PI punch and by 50% for the PO punch. It is worth mentioning that for PI punches, the correlation is rather linear, while for PO punches, nonlinearity starts to occur. Taking into consideration the stress level, the obtained values (Figure 19) suggest that even medium carbon steel should be able to carry such a load. However, it should be remembered that the load is a pulsating one and the contact pressure for such tools can exceed 75 MPa. For that reason, to obtain a satisfying tool life, high-speed tool steel should be used.

Although it is hard to find research to which the obtained results can be compared, since testing similar shapes has mainly been for medicine [6,7], the trend of decreasing the force for lower blade angles can be validated by the literature, such as in [28].

**Figure 20:**
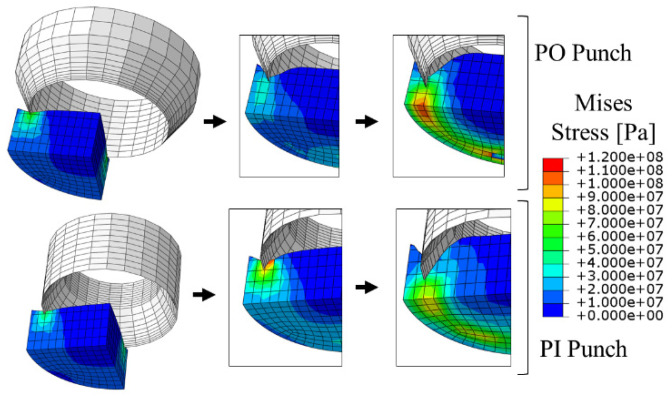
Phases of cutting in CEL FEM analyses for both PI and PO punches and belt TFL10S.

In Figure 20, the phases of the belt perforation modelling are presented for both types of punches. As can be observed, the behavior of the belt material is close to the real punching process, which proves that the CEL method is suitable for such an application.

## 4. Optimization of the Punch Geometry

In order to determine the optimization function, two indicators were used: indicator of the force, *x_F_*, and indicator of the stress, *x_S_*. A similar approach to tool optimization is presented in [11]. To derive these indicators, the following equations were used:

(3) $$x_{F}=F_{P}/\min\;\left(F_{P}\right)$$

(4) $$x_{S}=\sigma_{C}/\max\;\left(\sigma_{C}\right)$$

To determine the indicators, the opposite peak values are considered for each criterion (maximum stress and minimum punching force), and based on the obtained results, the reference values used in calculations will be taken for the lowest angle of the blade *β* in the analyzed range, which is 15°. The optimization function *f* is the product of both indicators, as presented below:

(5) $$f\left(\beta \right)=x_{F}\cdot x_{S}$$

**Figure 21:**
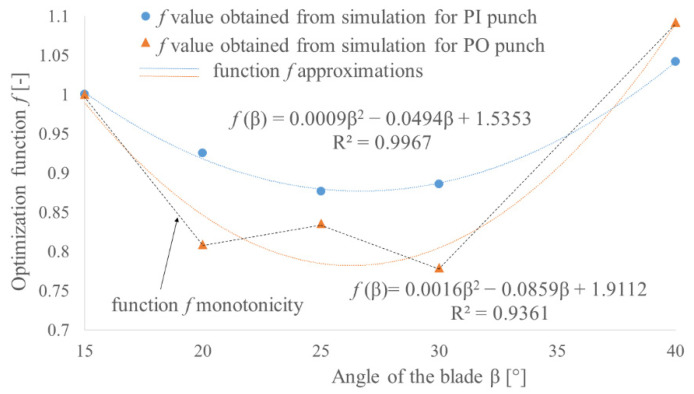
The optimization function for both PI and PO punches with diameter *d* = 10 mm and height of the blade *H* = 2.65 mm for belt TFL10S.

**Figure 22:**
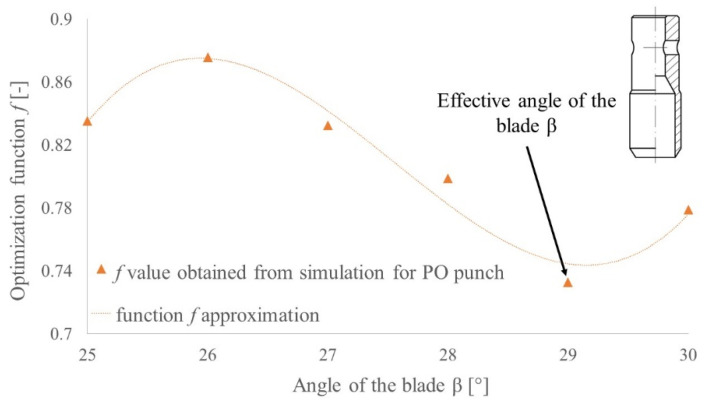
The optimization function for PO punch with diameter *d* = 10 mm and height of the blade *H* = 2.65 mm for belt TFL10S and for angle of the blade in range of 25°–30°.

In order to find the effective geometry of the punch, the optimization function should reach its minimum. For this value, the compromise between the force value and the stress in the punch is achieved. Figure 21 presents the approximations of optimization functions obtained from the FEM results for both PI and PO punches. In the case of the PI punch, the second-degree polynomial gives quite a good approximation, and this function reaches its minimum for 27.5°. This value will be treated as an effective one. In the case of the PO punch, the approximation with second-degree curve does not reflect the monotonicity of the optimization function (black dotted lines in Figure 21). Because of this, it does not provide the specific value of the effective angle of the blade *β*, but based on that, it can be concluded that the effective value lies in the range 25°–30°. In order to find specific value, additional FEM analyses for blade angles 26°, 27°, 28° and 29° were performed and their results are presented in Figure 22. Based on the presented data, the angle of the blade *β* = 29° can be treated as an effective one for PO punches.

**Figure 23:**
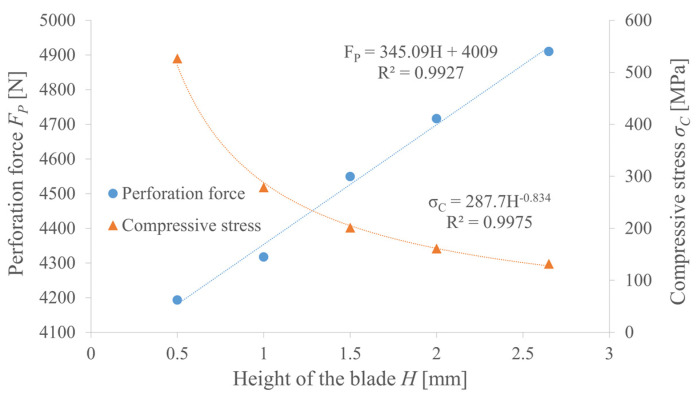
The influence of the height of the blade *H* on the perforation force *F_P_* and the punch compressive stress *σ_C_* for PI punch with diameter *d* = 10 mm and effective angle of the blade *β* = 27.5° mm for belt TFL10S (markers represent simulation results, lines represent approximations of functions *F_P_*(*H*) and *σ_C_*(*H*)).

In the above analysis, the height of the blade equals exactly the thickness of the belt; however, its reduction may also have an impact on the perforation force and punch stress. In the case of the PO punch, it is basically negligible, but for PI punches, the compression of the scrap generates high frictional resistance. As can be observed in Figure 23, there is a linear positive correlation between the height of the blade and the perforation force, but the force cannot be reduced by more than 15%. The nonlinear negative correlation between the blade height and the punch stress is much more significant since it can increase over five times.

It is also worth mentioning that values over 250 MPa, along with the pulsating load, may greatly reduce the tool life even when very tough steel is used. Based on that, the most advantageous and, as a result, the effective height of the blade should be close to the thickness of the belt—in this case, it is equal to 2.65 mm.

## 5. Conclusions

The analysis presented in this paper has shown that the hollow piercing punch with the cutting edge pointing outside (PO) is more advantageous than the one with the cutting edge pointing inside (PI) in terms of the punching forces and the tool stress level. However, for specific applications, the second tool can also be used.

Regardless of the type of punch, a positive correlation between both the angle and the height of the blade and the perforation force was proved in both experimental and simulation tests. The estimation functions presented in the paper can be successfully used to improve the design process of the punching machines. Based on the results, by selecting a proper tool, the force can even be reduced by 50%. For belt perforation, the effective blade angle equals 27.5° for the PI punch and 29° for the PO punch. In the case of the PI punch, the height of the blade should be maximized up to the thickness of the belt.

The presented research also proved that Coupled Eulerian-Lagrangian FEM analysis is a suitable method to model the punching process of polymer multilayer composite belts. The validation of the model presented in this paper showed that the belt perforation process is very complex and hard to model.

## Figures and Tables

**Table 1:** Material properties used in FEM modelling of the TFL10 belt.

Density *ρ* [kg/m^3^]	Young’s Modulus *E* [MPa]	Poisson Ratio *υ* [−]	Johnson–Cook Failure Model Parameters		
			*d*_1_ [−]	*d*_2_ [−]	*d*_3_ [−]
1140	235	0.2	−0.24	0.32	2.6

**Table 2:** Simulation parameters used in FEM modelling of the punching.

Analysis Time *t* [s]	Punch Velocity *v* [mm/s]	Friction Coefficient *μ* [−]	Maximum Displacement *x* [mm]	Yield Point *σ_plast_* [MPa]	
				PO Punch	PI Punch
55	50	0.6	2.75	80	120

**Table 3:** Summary of the experimental results for TFL10S belt.

Type of Punch	Diameter *d* [mm]	Angle of the Blade *β* [°]	Perforation Force *F_P_* [N]
PI	5	30	2455
	6	30	3008
	8	30	3238
	10	20	4567
		30	4916
		40	6750
PO	10	20	2397
		30	3272
		40	4778

## Data Availability

The data presented in this study are available on request from the corresponding author.
